# Leukotriene A4 Hydrolase and Hepatocyte Growth Factor Are Risk Factors of Sudden Cardiac Death Due to First-Ever Myocardial Infarction

**DOI:** 10.3390/ijms231810251

**Published:** 2022-09-06

**Authors:** Fredrik Landfors, Simon Vikström, Patrik Wennberg, Jan-Håkan Jansson, Jonas Andersson, Elin Chorell

**Affiliations:** 1 Department of Public Health and Clinical Medicine, Section of Medicine, Umeå University, 901 87 Umeå, Sweden; 2 Department of Public Health and Clinical Medicine, Skellefteå Research Unit, Umeå University, 901 87 Umeå, Sweden; 3 Department of Public Health and Clinical Medicine, Family Medicine, Umeå University, 901 87 Umeå, Sweden

**Keywords:** sudden cardiac death, circulating risk marker, plasma protein, myocardial infarction, plaque instability

## Abstract

Patients at a high risk for sudden cardiac death (SCD) without previous history of cardiovascular disease remain a challenge to identify. Atherosclerosis and prothrombotic states involve inflammation and non-cardiac tissue damage that may play active roles in SCD development. Therefore, we hypothesized that circulating proteins implicated in inflammation and tissue damage are linked to the future risk of SCD. We conducted a prospective nested case–control study of SCD cases with verified myocardial infarction (N = 224) and matched controls without myocardial infarction (N = 224), aged 60 ± 10 years time and median time to event was 8 years. Protein concentrations (N = 122) were measured using a proximity extension immunoassay. The analyses revealed 14 proteins significantly associated with an increased risk of SCD, from which two remained significant after adjusting for smoking status, systolic blood pressure, BMI, cholesterol, and glucose levels. We identified leukotriene A4 hydrolase (LTA4H, odds ratio 1.80, corrected confidence interval (CI_corr_) 1.02–3.17) and hepatocyte growth factor (HGF; odds ratio 1.81, CI_corr_ 1.06–3.11) as independent risk markers of SCD. Elevated LTA4H may reflect increased systemic and pulmonary neutrophilic inflammatory processes that can contribute to atherosclerotic plaque instability. Increased HGF levels are linked to obesity-related metabolic disturbances that are more prevalent in SCD cases than the controls.

## 1. Introduction

Sudden cardiac death (SCD) poses a challenging problem for preventive cardiology and accounts for more than one-half of all cardiac deaths [1]. As most SCD occurs without warning symptoms in individuals with a low estimated risk of cardiovascular disease (CVD), effective risk-reduction strategies beyond traditional risk factors are needed. Primary prevention is the desirable option because the prognosis of sudden out-of-hospital cardiac arrest is poor, with a survival rate of approximately 10% [2]. A proposed strategy is to augment current clinical risk models with new informative risk markers [3,4,5].

Atherosclerotic coronary heart disease is the major cause of SCD after 35 years of age [6]. The specific mechanisms that tip the scale from coronary disease survival to triggering fatal myocardial infarction are unknown. The fatal events may be triggered by altered pathogenic factors, including an atherosclerotic plaque together with disturbed hemostasis, electrolyte imbalances, inflammation, and/or non-cardiac organ dysfunction [3,5,7]. To improve clinical risk models, we can combine traditional CVD risk factors with new markers of inflammation and organ dysfunction. In addition, developments in laboratory science have made it possible to measure hundreds of proteins using small volumes of blood [8]. Circulating plasma proteins are either actively secreted from cells or, more interestingly, leaked from tissues and interstitial cellular processes. For example, cytokines may leak into the bloodstream as a result of local inflammation or apoptosis/necrosis in injured tissues [9]. Thus, circulating proteins are of major interest in SCD research as potential risk markers and to highlight underlying disease processes to identify potential targets for prevention.

We hypothesized that a set of circulating proteins associated with inflammation and tissue injury were altered in individuals who later died of SCD. To the best of our knowledge, no previous study has evaluated a broader set of inflammatory markers or circulating markers that may be indicative of non-cardiac tissue damage in a population-based study of SCD.

## 2. Results

### 2.1. Baseline Characteristics

**Figure 1:**
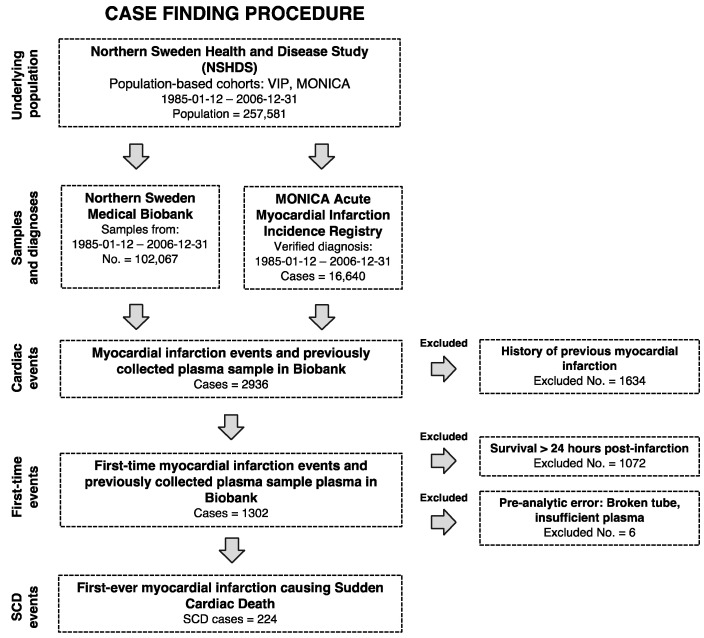
Procedure for identifying cases. MONICA: World Health Organization′s Monitoring of Trends and Determinants in Cardiovascular Disease study.

During a median follow-up of 8 years, we identified 224 SCD cases and 224 controls with available plasma (Figure 1).

The median age at the time of blood sampling was 60 years and the median time to an event was 8 years. In total, 108 (48.2%) SCD cases died within 1 h of first symptom onset. For 116 cases (51.8%), death was likely to have occurred within 24 h after first symptom onset based on death certificates and medical records. Twenty-seven (12.1%) SCD cases had ECG signs of ST-elevation myocardial infarction before death. Six cases (2.6%) had ECG signs and/or cardiac enzyme elevation that met non-ST-elevation myocardial infarction criteria. One (0.4%) case had isolated LBBB and 190 cases (84.8%) were not evaluated by ECG or cardiac enzymes before death. The SCD cases had higher total cholesterol, BMI, fasting glucose, and systolic and diastolic blood pressure. SCD cases were more likely to be current smokers and have a history of diabetes (Table 1). Relatively few women (20.5%) suffered SCD during the study. Due to a lack of statistical power, it was not possible to conduct sex-stratified analyses.

### 2.2. Plasma Proteins and SCD Risk

The conditional model without adjustments for traditional risk factors revealed 14 proteins that were associated with incident SCD: hepatocyte growth factor (HGF), leukotriene A4 hydrolase (LTA4H), kidney injury molecule 1 (KIM1), osteoprotegerin (OPG), fibroblast growth factor 23 (FGF23), oncostatin M (OSM), CUB domain-containing protein 1 (CUB1), platelet-derived growth factor C (PDGFC), interleukin 18 (IL18), vascular endothelial growth factor A (VEGFA), calcitonin-related polypeptide alpha (CALCA), interleukin 6 (IL6), TNF superfamily member 14 (TNFSF14), and interleukin 18 receptor 1 (IL18R1) (Table 2). LTA4H and HGF remained independently associated with the risk of SCD after adjusting for systolic blood pressure, BMI, fasting glucose, smoking status, and total cholesterol (Table 2). A sensitivity analysis of the model adjustments showed a dose–response relationship between the incremental adjustment and reduction of the effect estimates in all risk markers except LTA4H (Figure S1 in the Supplementary Materials). The stratification analysis revealed that the estimated effects were relatively stable across subphenotypes. Crude odds ratio estimates remained within the range of 1.5–2.0 and 2.0–2.5 range for LTA4H and HGF, respectively, when stratifying for time to death from first symptom onset, time-to-event, ECG classification, chronic coronary syndrome medication, and diabetes status (Figure S2 in the Supplementary Materials). All of the measured risk markers showed an approximate linear relationship between the logarithm of the predicted odds and the predictor values (Figure S3 in the Supplementary Materials).

### 2.3. Plasma Proteins and Traditional Cardiovascular Disease Risk Factors

**Figure 2:**
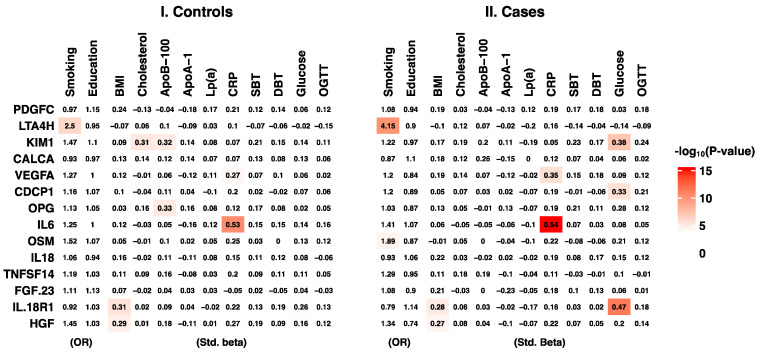
Risk factors vs. protein levels. Heat map of linear or logistic regression effect estimates (standardized β (Std. beta) for continuous variables/odds ratios (ORs) for categorical variables) adjusted for age and sex, and stratified by case/control status. The control group was selected from the general population, providing population estimates.

Adjusting for classical cardiovascular risk factors decreased the effect size estimates for most proteins (Table 2). To investigate whether strong clinical risk factor-confounding effects were present, we performed a secondary analysis of protein concentration associations and traditional cardiovascular risk factors. As the cases and controls had different risks for CVD, we decided to model them separately. The analyses revealed eight protein–risk factor associations in the control group and nine associations in the case group (Figure 2). Five of the 17 protein-risk factor associations were concordant between cases and controls: LTA4H with cigarette smoking, HGF with BMI, IL18R1 with BMI, VEGFA with CRP, and IL6 with CRP.

## 3. Discussion

The present study showed an independent and positive association between circulating LTA4H and the risk of SCD for the first time. LTA4H has previously been linked with atherosclerotic plaque inflammation and instability, which is a common trait of fatal coronary thrombus [12]. Our secondary analysis linked LTA4H to inflammation in smoke-exposed lungs, which is consistent with previous literature [13,14,15]. We also showed an independent and positive association between SCD and HGF, which has previously been linked to obesity and CVD [16,17]. Furthermore, we report 12 additional inflammation and tissue damage-linked proteins that were associated with SCD when adjusting for age and sex.

The LTA4H enzyme has both pro-inflammatory and anti-inflammatory activity [18]. The pro-inflammatory function of this enzyme was first discovered when the downstream effects of arachidonic acid in the lipoxygenase pathway were mapped [19,20,21]. In this pathway, leukocytes use the LTA4H hydrolase activity to convert leukotriene A4 to the pro-inflammatory leukotriene B4 [22]. To this end, experimental studies have linked imbalances in the gene expression of enzymes that regulate the production of pro-inflammatory leukotrienes with plaque instability [13]. Thus, our observation of increased LTA4H in SCD cases may be a result of increased formation of vulnerable plaques.

In contrast, experimental studies have also shown an anti-inflammatory effect of LTA4H in the lungs of smokers. Here, LTA4H aminopeptidase acts in an anti-inflammatory manner when it degrades the pro-inflammatory proline–glycine–proline oligopeptides that otherwise accumulate during chronic smoke inhalation [14,15]. Consistent with previous findings [23], circulating LTA4H levels were independently associated with active smoking in our study (Figure 2). As the lungs are well-perfused, it would not be surprising if plasma LTA4H reflects leakage from smoke-induced pulmonary inflammation. This argument is strengthened by the findings that LTA4H protein was highly expressed in the lungs compared to other tissues [11], and LTA4H is primarily thought to be active inside immune cells and is not predicted to be actively secreted into plasma (Table 2). However, the estimated effect of LTA4H did not decrease in our fully adjusted model, suggesting that independent effects still exist. This may represent a pathway of atherothrombotic inflammation. In favor of this reasoning, a previous study found that LTA4H gene expression was increased in human plaques, and that the RNA expression was positively associated with clinical symptoms of plaque instability [13]. LTA4H inhibitors are currently being developed for possible future treatments [24]. We suggest that LTA4H is a potential risk marker for SCD. However, both the discriminatory ability and pathophysiological interpretation of elevated circulating LTA4H need to be evaluated in larger cohort studies, preferably with measures of the presence and progression of atherosclerotic plaques.

As mentioned previously, we also showed an independent association between HGF and an increased SCD risk. HGF is a growth factor of mesenchymal origin that regulates mitogenesis, growth, morphogenesis, and motility at the cellular level [25,26]. Previous studies have shown that elevated HGF levels are independently associated with increased CVD risk [16,17]. Although seemingly related to plasma lipids, Mendelian randomization studies have placed circulating HGF on the causal pathway from BMI [27]. In the present study, we found similar associations with both SCD and BMI (Table 2 and Figure 2). In a previous study, we showed that BMI and diabetes are associated with an increased risk of future SCD compared to survivors of myocardial infarction [28]. This suggests that plasma HGF is elevated by the metabolic disturbances that are more prevalent in incident SCD cases. As these metabolic disturbances may not be perfectly measured by BMI, this potential confounding relationship may explain why SCD cases in this study had higher levels of HGF.

Many of the 12 additional proteins that were linked to incident SCD in the crude model (KIM1, OPG, FGF23, OSM, CDCP1, PDGFC, IL18, VEGFA, CALCA, IL6, TNFSF14, and IL18R1) are well described within the context of CVD. However, it is not within the scope of this article to comment on all of them in detail. A genetics-informed meta-analysis of >30 000 individuals suggested causal pathways both to and from cardiometabolic and inflammatory disease [27]. Notably, some of the SCD-associated proteins, such as KIM1, showed different correlation patterns with traditional risk factors in control subjects and SCD cases in the present study. Circulating KIM1 was associated with total cholesterol and ApoB-100 in controls, but was mainly associated with fasting glucose levels in SCD cases (Figure 2). These analyses are to be considered highly explorative. However, previous epidemiological studies and biological plausibility indicate that some of the differential correlations observed in cases and controls may be true. Plasma KIM1 has been associated with both lipid metabolism and kidney damage, including diabetic nephropathy [27,29,30]. As diabetes was overrepresented in our case group (Table 1), it is not unlikely that the differential correlation patterns reflect a higher burden of diabetic nephropathy in SCD victims. This also reflects another, more general, question about the statistical modelling of proteomics and metabolomics data. It is not clear whether it is more biologically relevant to model differential interaction networks rather than compare averages as in our study. Currently, there are interesting methodological advances being made, such as comparing the differences in protein–protein correlation structures between cases and controls [31].

Clinically informative risk markers must have a high prognostic accuracy or be easily targeted by low-cost interventions in order to provide clinical utility in low-absolute risk populations. During large parts of the study period, the annual incidence for 35 to 64-year-olds was 65 per 100,000 persons for men and 15 per 100,000 persons for women [32]. Although the case sample size is comparable to several previous studies of other SCD risk markers [33,34,35], it was not feasible to develop a risk prediction model. The risk markers identified in this study should be included in future collaborative projects with larger samples.

A major strength of this study was that the case–control design was nested within a population-based cohort. This means that blood was sampled several years before the event. The parent cohort participation rates were high. In addition, we were able to include subjects who died of out-of-hospital cardiac arrest and for whom no resuscitation was attempted. These individuals are rarely included in registries available for research. We also conducted rigorous matching for several pre-analytical and demographic variables.

As the study was observational, causal interpretation of the effect estimates (Table 2, Figure 2) should be cautious because conclusions cannot be drawn about the true effects from the regression model effect size estimates alone. Whether the observed reduction in estimated effect sizes when adjusting for traditional risk factors (Table 2) was a result of confounding by cardiovascular risk factors or caused by imprecision due to low statistical power cannot be determined within this study. Even though we excluded individuals with previous myocardial infarction, we were not able to exclude subjects with a previous diagnosis of chronic coronary syndrome. Future studies should include myocardial infarction survivors as controls to provide information on the clinical course and prognosis. The study was performed in a population that is quite genetically homogenous and distinct [36]. As plasma protein levels often are stable over time [37] and inter-individual variation can be explained at least partially by heritability [38], the results cannot yet be generalized to other populations. However, population-based cohorts of SCD with verified myocardial infarction and no previous cardiovascular events are rare, which makes validation studies difficult to conduct.

## 4. Materials and Method

### 4.1. Study Population

We conducted a prospective nested case–control study derived from the Västerbotten Intervention Program (VIP) and World Health Organization’s Monitoring of Trends and Determinants in Cardiovascular Disease (MONICA) studies in the counties of Västerbotten and Norrbotten in northern Sweden. The VIP and MONICA studies had participation rates of 65% and 77%, respectively. Case registration in MONICA included seven district general hospitals and one university hospital. VIP began in 1985 and is a population-based screening and intervention program integrated at the primary health care level through which inhabitants turning 40, 50 and 60 years old in Västerbotten county are invited for health screening and a health dialogue with a trained nurse [39]. The MONICA study also started in 1985 and invited 2500 randomly selected individuals aged 25–74 years for risk factor surveys every 5 years. The screening procedures for both VIP and MONICA involve a health examination and extensive questionnaire [39,40] that includes questions concerning different drug treatments, smoking habits, level of education, and prior diagnoses, such as diabetes. Participants are requested to donate fasting blood samples for research purposes, and these samples are stored at −80 °C. Within the MONICA event registry, all myocardial infarctions in Västerbotten and Norrbotten county were registered according to standardized MONICA criteria [40]. Thus, the event registration for the VIP and MONICA cohorts included in this study are standardized according to the same protocols. The event registration procedure is based on general practitioner reports, hospital discharge records, necropsy reports, and death certificates. For information on deaths in the community, death certificates with ICD codes I41.0–I41.4 were collected and validated according to the MONICA criteria. Therefore, we were able to include those who died of cardiac events out-of-hospital even when no resuscitation was attempted. All prospective cases with a verified first myocardial infarction in the VIP and MONICA studies between 1986 and 2006 were identified in the MONICA registry. We defined SCD as a sudden unexpected death within 24 h from first-ever myocardial infarction. Methods for classification and diagnosis are described in detail elsewhere [41]. Thus, the study population was obtained from the MONICA event registry by identifying all individuals with definite or possible myocardial infarction causing SCD who had participated in VIP or MONICA. A total of 224 individuals were identified (Figure 1). Case–control matching was performed in a 1:1 ratio. Controls without myocardial infarction that met the matching criteria and had frozen plasma available were randomly selected from the parent cohort. Matching criteria were sex, age at blood sample collection (±2 years), sample date (±4 months), type of health examination (VIP or MONICA), and number of previous plasma sample freeze–thaw cycles. Propensity score matching was used for geography and patient self-reported fasting time prior to blood sample collection.

### 4.2. Baseline Variables

BMI was calculated as weight in kilograms divided by the squared height in meters. Blood pressure was measured in a sitting position. We dichotomized smoking status into smoker or non-smoker, for which occasional smokers were considered non-smokers. Diabetes was defined by self-reported use of diabetes medication or a known diagnosis of diabetes as reported in the questionnaire or based on fasting blood glucose.

### 4.3. Blood Sample Collection and Storage

Blood samples were collected between 1986 and 2006 by trained medical personnel at local primary care units according to standardized protocols [39]. The study participants were instructed to fast overnight or over forenoon, and 95% of participants complied (Table 1). After collection, the plasma samples were aliquoted into Eppendorf tubes and stored in a −80 °C freezer at the Northern Sweden Medical Biobank. Total cholesterol and plasma glucose levels were analyzed shortly after the blood draw using a bench top instrument (Reflotron^®^; Roche Diagnostics GmbH, Mannheim, Germany). For samples collected in 2005 and 2006, a Hemocue capillary glucose measurement instrument was used (Quest diagnostics, Secaucus, NJ, USA).

ApoA-1, ApoB-100, and CRP concentrations were analyzed using Tina-quant kits (APOAT, APOBT, CRPL3; Roche Diagnostics GmbH, Mannheim, Germany).

### 4.4. Proximity Extension Assay

Protein concentrations were measured using proximity extension assays (Organ damage v.3311, inflammation v.3021; Olink proteomics, Uppsala, Sweden). Briefly, a solution containing DNA polymerase and protein-specific antibodies, each linked to a DNA strand, was added to the plasma samples on a 96-well plate. When a pair of the protein-specific antibodies bound to the same protein, an analyte-specific reporter sequence was created. The DNA polymerase then transcribed the by-proximity-ligated DNA strands. Relative protein concentrations were measured by quantitative PCR. As antibody-linked DNA strand proximity is required for PCR amplification, the measurement error from antibody cross-reactivity is reduced [42]. Protein concentrations were reported as log_2_-transformed normalized protein expression (NPX) values. The NPX was determined by correcting the analyte threshold cycle (Ct value) for extension and amplification/detection effects, interplate variation, and negative control Ct values. Case–control pairs were analyzed in neighboring wells. Quality control showed that potentially confounding pre-analytical and traditional CVD risk factors were well randomized between plates (Online Supplement). NPX values measured on the log_2_ scale have a sigmoid (S-curve) relationship with the absolute protein concentration. Thus, for values within the linear range, 1-NPX meant doubling of the protein concentration. However, for values outside the limit of detection (LOD), the relationship with the true concentration can be non-linear, which may bias estimates. Therefore, we excluded proteins with >5% of measurements outside the LOD from further analysis. For proteins with <5% of values within the LOD, we used the raw NPX value in downstream analyses. The targeted proteomics assay detected 73 out of 92 proteins in the organ damage panel, and 67 out of 92 proteins in the inflammation panel. Thus, the rates of detection were 79% and 73%, respectively. These rates were above the manufacturer’s expected detectability in EDTA plasma (>75% and >70%). Eighteen additional proteins had >5% of measurements outside the LOD range. The proportion of measurements outside the LOD for each protein is shown in the Supplementary Materials (Tables S1–S3 in the Supplementary Materials).

### 4.5. Statistical Analysis

In our main analysis, effect estimates are reported as the change in odds ratio (OR) per one standard deviation (1-SD) of the change in NPX. A separate conditional logistic regression model was fitted for each plasma protein using the matching set ID for stratum adjustment. When determining the statistical threshold for significance according to a family-wise error rate of 5%, we used the Bonferroni correction method. The threshold was calculated as $P_{thresh}=\frac{0.05}{122}\approx 4.1\times 10^{-4}$. To test whether risk markers had independent associations, we fit conditional logistic regression models adjusted for fasting glucose, total cholesterol, smoking status, systolic blood pressure, and body mass index (BMI). Some of the variables used to adjust the fully adjusted model contained missing values (range: 1.3–9.6%; Tables S1–S3 in the Supplementary Materials). These were imputed by predictive mean matching in multivariate imputation by chained equations [43]. The parent cohort was used to fit the imputation model. In the secondary analysis of clinical risk markers and proteins, logistic or linear regression models were fitted with adjustments for age and sex. To deal with the selection effect that can result in biased estimates when performing a secondary analysis of a case–control study [44], we performed separate analyses for cases and controls. The Bonferroni correction for the secondary analysis was calculated as $P_{thresh}=\frac{0.05}{168}\approx 3.0\times 10^{-4}$. Proteins were annotated for Gene Ontology molecular function, predicted location, and RNA tissue enrichment by querying The Human Protein Atlas (v. 20.0) using UniProt identifiers [10]. Protein tissue specificity (TS) was defined as proteins with a TS score > 4.0 as determined by Jiang et al. [43]. The statistical analyses were performed using R (v. 4.0.2). Full documentation with code, as well as a quality control protocol, is available in an online supplement (http://github.com/fredlandfors/FIA3-SCD-protf (accessed on 12 February 2020).

## 5. Conclusions

This population-based study shows that LTA4H and HGF are associated with an increased risk of SCD due to MI. The findings may help improve risk models to identify individuals at risk of SCD and increase our understanding of the mechanisms underlying SCD, making new preventive interventions possible.

## Figures and Tables

**Table 1:** Baseline characteristics of the study populations.

	N_OBS_	Cases	N_obs_	Cases	Controls
**Age at blood sampling (years)**	224	59.8 (IQR: 50.1–60.1; range: 29.8–69.9)	224	59.9 (IQR: 50.2–60.2; range: 30.2–72.9)	224
**Age at event (years)**	224	64.6 (IQR: 58.7–68.7; range: 40.6–74.9)			224
**Age at blood sampling (years)**	224	59.8 (IQR: 50.1–60.1; range: 29.8–69.9)	224	59.9 (IQR: 50.2–60.2; range: 30.2–72.9)	224
***Sex***	*224*		*224*		*224*
**Male**		178 (79.5%)		178 (79.5%)	
**Female**		46 (20.5%)		46 (20.5%)	
**Serum total cholesterol (mmol/L)**	216	6.43 ± 1.32	214	6.09 ± 1.14	216
**Apolipoprotein B-100 (g/L)**	170	1.32 ± 0.292	173	1.17 ± 0.254	170
**Apolipoprotein A-1 (g/L)**	169	1.36 ± 0.211	173	1.42 ± 0.255	169
**C-reactive protein (mg/L)**	168	1.7 (IQR: 0.788–3.39; range: 0–61.8)	169	0.9 (IQR: 0.47–2.01; range: 0.03–47.9)	168
**Lipoprotein(a) (μmol/L)**	169	17.7 (IQR: 7.1–69.3; range: 0.9–398)	173	13.7 (IQR: 6.7–33.1; range: 1.9–301)	169
**Body mass index (kg/m^2^)**	223	27.1 (IQR: 25.2–29.7; range: 19.9–44.8)	219	25.6 (IQR: 23.2–27.8; range: 17.9–40.7)	223
**Systolic blood pressure (mmHg)**	220	142 ± 20	220	133 ± 18	220
**Diastolic blood pressure (mmHg)**	220	86.7 ± 10.9	220	82.3 ± 10.2	220
**Glucose (mmol/L)**	201	5.5 (IQR: 5.05–6.2; range: 3.9–26.9)	204	5.4 (IQR: 5.09–5.9; range: 2.9–12.5)	201
***Current smoker***	*218*		*213*		*218*
**Yes**		82 (37.6%)		50 (23.5%)	
**No**		136 (62.4%)		163 (76.5%)	
***Diabetes***	*223*		*218*		*223*
**Yes**		23 (10.3%)		5 (2.3%)	
**No**		200 (89.7%)		213 (97.7%)	
***Secondary education***	*209*		*212*		*209*
**Yes**		92 (44%)		125 (59%)	
**No**		117 (56%)		87 (41%)	
***Time to death ^1^***	*224*				*224*
**<1 h**		108 (48.2%)			
**<24 h**		116 (51.8%)			
***Blood pressure lowering***	*216*		*204*		*216*
**Yes**		65 (30.1%)		25 (12.3%)	
**No**		151 (69.9%)		179 (87.7%)	
***ASA or nitroglycerin***	*216*		*204*		*216*
**Yes**		24 (11.1%)		10 (4.9%)	
**No**		192 (88.9%)		194 (95.1%)	
***Lipid-lowering drug***	*177*		*173*		*177*
**Yes**		19 (10.7%)		1 (0.6%)	
**No**		158 (89.3%)		172 (99.4%)	

N_obs_: number of complete observations per variable. Data are reported as n (%), mean ± standard deviation, or median with interquartile range (IQR) and range. ^1^ Estimated time to death from symptom onset.

**Table 2:** Proteins associated with increased SCD risk.

Protein	Gene	Molecular Function ^1^	Predicted Location ^2^	Enhanced Tissue Expression ^3^	OR (95% CI) ^4^
Hepatocyte growth factor	***HGF*** (*DFNB39*, *F-TCF*, *HGFB*, *HPTA*, *SF*)	Growth factor, serine protease homolog	Secreted	*RNA:* Placenta *Protein:* Tibial and coronary arteries	**2.27 (1.39–3.71)**
Leukotriene A4 hydrolase	***LTA4H***	Hydrolase, metalloprotease, protease	Intracellular	*RNA:* Low tissue specificity *Protein:* Lung	**1.67 (1.09–2.55)**
Kidney injury molecule 1 (Hepatitis A virus cellular receptor 1)	***KIM1*** (*CD365*, *HAVCR*, *HAVCR1*, *HAVCR-1*, *TIM-1*, *TIM1*, *TIMD1*)	Host cell receptor for virus entry, receptor	Intracellular, membrane	RNA: Kidney	**2.11 (1.35–3.32)**
Osteoprotegerin (TNF receptor superfamily member 11b)	***OPG*** (*OCIF*, *TR1*, *TNFRSF11B*)	Receptor	Secreted	*RNA:* Thyroid, kidney *Protein:* Aorta, tibial and coronary arteries	**1.76 (1.17–2.66)**
Fibroblast growth factor 23	***FGF23***	Growth factor	Secreted	*RNA:* Blood, heart muscle, liver, urinary bladder	**1.69 (1.07–2.65)**
Oncostatin M	***OSM*** (*MGC20461*)	Cytokine, mitogen	Intracellular, secreted	*RNA:* Blood, bone marrow	**1.57 (1.08–2.27)**
CUB domain-containing protein 1	***CDCP1*** (*CD318*, *SIMA135*)		Intracellular, membrane	*RNA:* Low tissue specificity	**1.56 (1.05–2.31)**
Platelet-derived growth factor C	***PDGFC*** (*fallotein*, *SCDGF*)	Developmental protein, growth factor, mitogen	Secreted	*RNA:* Low tissue specificity Protein: Tibial artery and aorta	**1.66 (1.06–2.59)**
Interleukin 18	***IL18*** (*IGIF*, *IL-18*, *IL-1g*, *IL1F4*)	Cytokine	Intracellular, secreted	*RNA:* Skin, esophagus Protein: Skin, esophagus	**1.57 (1.05–2.35)**
Vascular endothelial growth factor A	***VEGFA*** (*VEGF*, *VEGF-A*, *VPF*)	Developmental protein, growth factor, heparin-binding, mitogen	Intracellular, secreted	*RNA:* Low tissue specificity	**1.66 (1.11–2.5)**
Calcitonin-related polypeptide alpha	***CALCA*** (*CALC1*)	Hormone	Secreted	*RNA:* Thyroid, parathyroid	**1.59 (1.02–2.47)**
Interleukin 6	**IL6** (*BSF2*, *HGF*, *HSF*, *IFNB2*, *IL-6*)	Cytokine, growth factor	Intracellular, secreted	*RNA:* Adipose, lymphoid tissue	**1.54 (1.03–2.29)**
TNF superfamily member 14	***TNFSF14*** (*CD258*, *HVEM-L*, *LIGHT*, *LTg*)	Cytokine	Intracellular, membrane, secreted	*RNA:* Blood, liver	**1.5 (1.03–2.2)**
Interleukin 18 receptor 1	***IL18R1*** (*CD218a*, *IL-1Rrp*, *IL1RRP*)	Hydrolase, receptor	Intracellular, membrane	*RNA:* Lung	**1.54 (1.07–2.21)**
Hepatocyte growth factor	***HGF*** (*DFNB39*, *F-TCF*, *HGFB*, *HPTA*, *SF*)	Growth factor, serine protease homolog	Secreted	*RNA:* Placenta *Protein:* Tibial and coronary arteries	**2.27 (1.39–3.71)**
Leukotriene A4 hydrolase	***LTA4H***	Hydrolase, metalloprotease, protease	Intracellular	*RNA:* Low tissue specificity Protein: Lung	**1.67 (1.09–2.55)**
Kidney injury molecule 1 (Hepatitis A virus cellular receptor 1)	***KIM1*** (*CD365*, *HAVCR*, *HAVCR1*, *HAVCR-1*, *TIM-1*, *TIM1*, *TIMD1*)	Host cell receptor for virus entry, receptor	Intracellular, membrane	RNA: Kidney	**2.11 (1.35–3.32)**
Osteoprotegerin (TNF receptor superfamily member 11b)	***OPG*** (*OCIF*, *TR1*, *TNFRSF11B*)	Receptor	Secreted	*RNA:* Thyroid, kidney *Protein:* Aorta, tibial and coronary arteries	**1.76 (1.17–2.66)**
Fibroblast growth factor 23	***FGF23***	Growth factor	Secreted	*RNA:* Blood, heart muscle, liver, urinary bladder	**1.69 (1.07–2.65)**
Oncostatin M	***OSM*** (*MGC20461*)	Cytokine, mitogen	Intracellular, secreted	*RNA:* Blood, bone marrow	**1.57 (1.08–2.27)**
CUB domain-containing protein 1	***CDCP1*** (*CD318*, *SIMA135*)		Intracellular, membrane	*RNA:* Low tissue specificity	**1.56 (1.05–2.31)**
Platelet-derived growth factor C	***PDGFC*** (*fallotein*, *SCDGF*)	Developmental protein, growth factor, mitogen	Secreted	*RNA:* Low tissue specificity Protein: Tibial artery and aorta	**1.66 (1.06–2.59)**

^1^ Gene ontology (GO) molecular function retrieved from Human Protein Atlas v. 20.0 (HPA20) [10]. The most common gene annotation is shown in bold and synonyms in brackets. ^2^ Secretome location from HPA20. ^3^ RNA expression enrichment was retrieved from HPA20. Protein tissue specificity scores were retrieved from Jiang et al. (GTEx) [11]. ^4^ Adjusted for age, sex, blood draw date (±4 months), freeze–thaw cycles, fasting status, and geography. Adjusted for crude model factors and body mass index, fasting glucose, smoking status, total cholesterol, and systolic blood pressure. All reported 95% CIs are corrected for multiple comparisons (n_comparisons_ = 122). *p*-values are reported without correction. OR: Odds ratio. CI: Confidence interval.

## Data Availability

Full documentation with code, as well as a quality control protocol, is available in an online supplement (www.github.com/fredlandfors/FIA3-SCD-prot).

## Supplementary Materials

The following supporting information can be downloaded at: https://www.mdpi.com/article/10.3390/ijms231810251/s1.
