# Supplementary material for: Leukotriene A4 Hydrolase and Hepatocyte Growth Factor Are Risk Factors of Sudden Cardiac Death Due to First-Ever Myocardial Infarction
Source: Int J Mol Sci. 2022 Sep 6;23(18):10251. doi: 10.3390/ijms231810251 (PMC9499415; doi:10.3390/ijms231810251)
Supplement: Supplementary file 1 [file ijms-23-10251-s001.zip › Supplemental Material.pdf]

Supplemental Material

SUPPLEMENTARY FIGURE S1

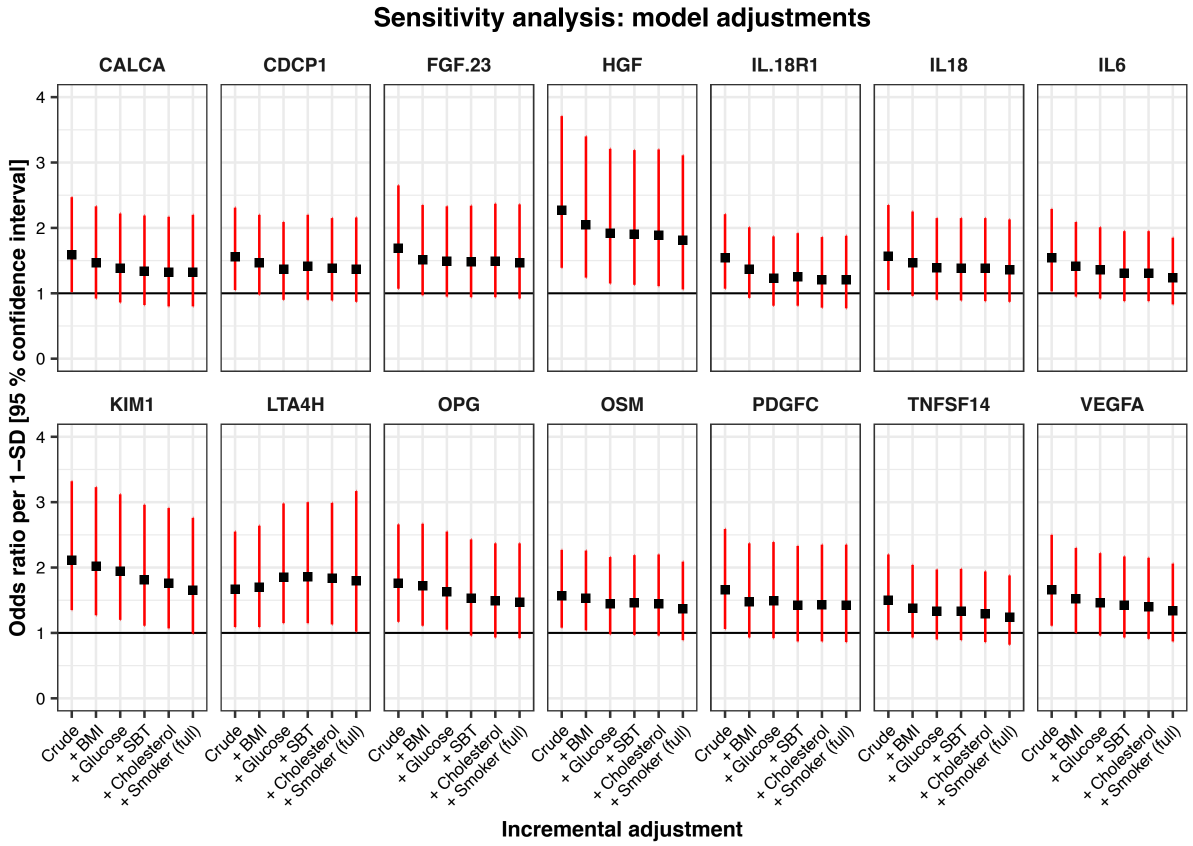

Figure S1. Sensitivity analysis of model adjustments. Effect estimates with 95% confidence intervals are shown for proteins using conditional logistic regression with incremental adjustment for traditional cardiovascular disease risk factors. The 95% confidence intervals were corrected for multiple comparisons ($n_{comparisons}$ = 122).

SUPPLEMENTARY FIGURE S2

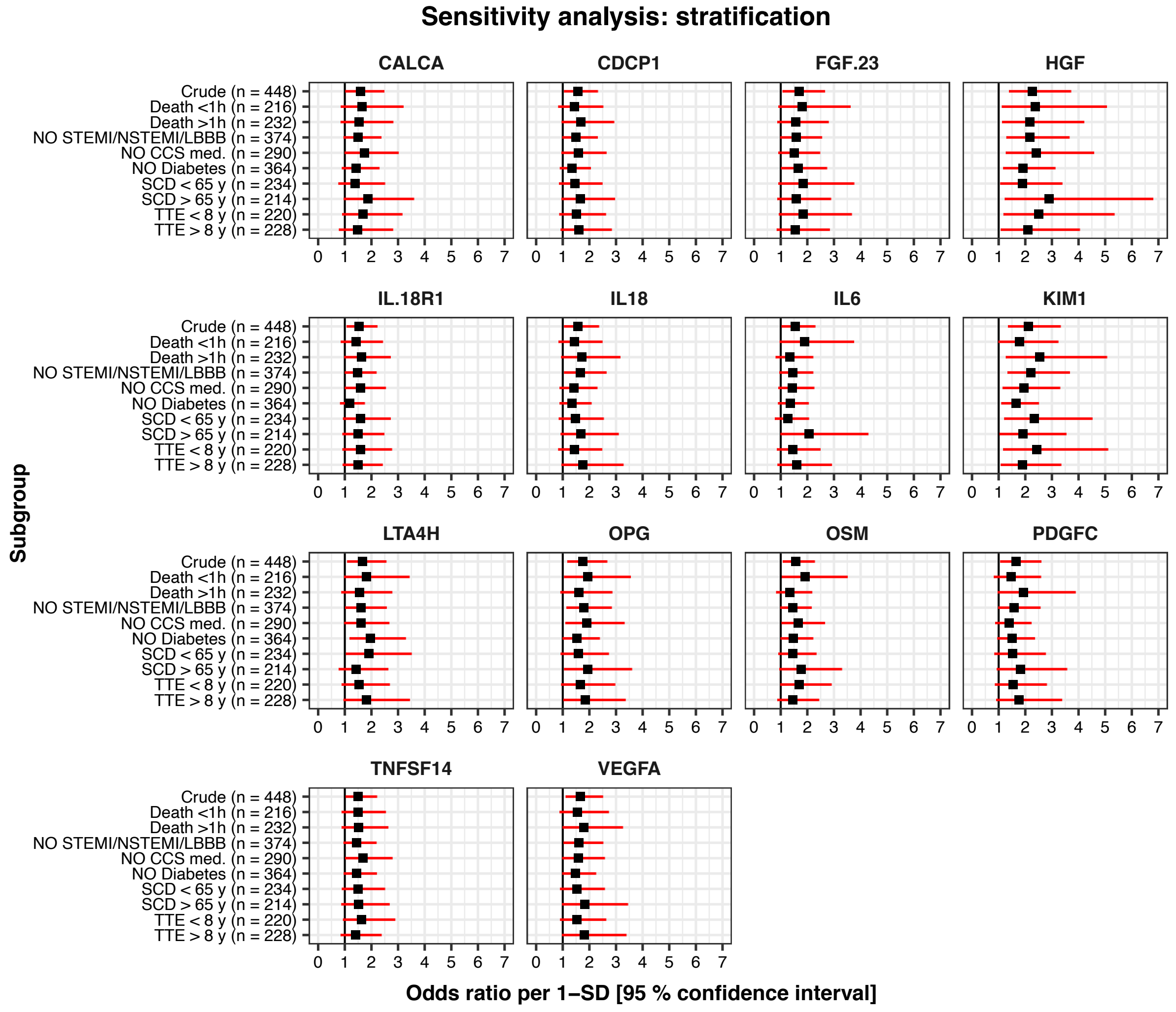

Figure S2. Sensitivity analysis for subgroup stratification. Effect estimates with 95% confidence intervals are shown for proteins using conditional logistic regression with stratification for different phenotypic subgroups. Each conditional logistic regression model was adjusted for age, sex, and preanalytical variables. The 95% confidence intervals were corrected for multiple comparisons ($n_{comparisons}$ = 122).

SUPPLEMENTARY FIGURE S3

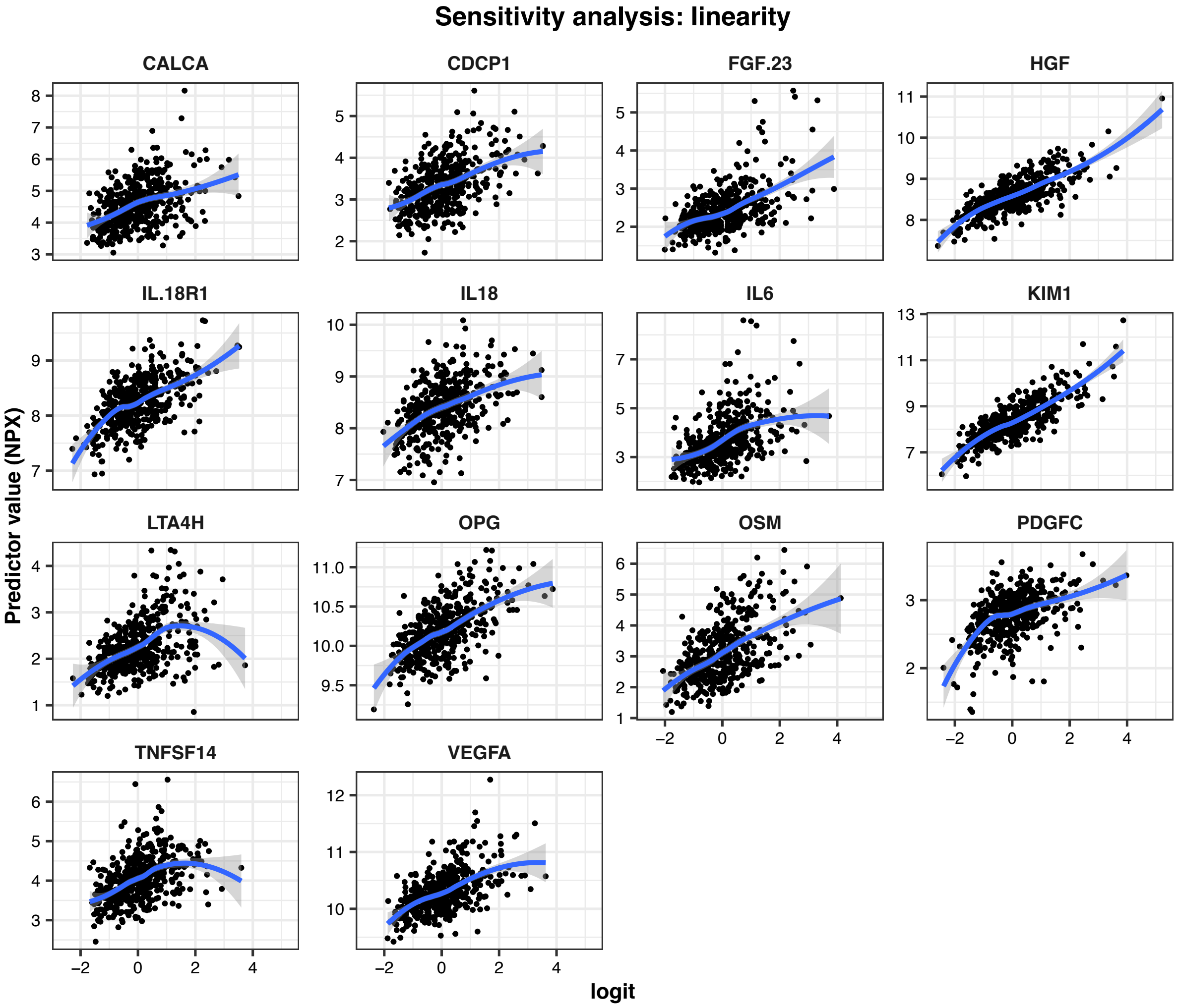

Figure S3. Sensitivity analysis for the assumption of linearity. The logarithm of the odds is plotted against normalized protein expression (NPX). Each logistic regression model was adjusted for age, sex, smoking status, total cholesterol, systolic blood pressure, body mass index, fasting blood glucose, and preanalytical variables.
